# Endoscopic Ultrasonography-Guided Drainage of the Pancreatic Duct (EUS-PD)—Indications and Results with a Literature Review

**DOI:** 10.3390/jcm13247709

**Published:** 2024-12-17

**Authors:** Uwe Will, Frank Fueldner, Theresa Buechner, Frank Meyer

**Affiliations:** 1 Department of Gastroenterology, Hepatology and General Internal Medicine, Municipal Hospital (“SRH Wald-Klinikum”), Str. des Friedens 122, 07548 Gera, Germany; frank.fueldner@srh.de (F.F.); theresa.buechner@srh.de (T.B.); 2 Department of General, Abdominal, Vascular and Transplant Surgery, Otto-von-Guericke University with University Hospital, Leipziger Str. 44, 39120 Magdeburg, Germany

**Keywords:** EUS-guided pancreatic duct drainage [EUS-PD], retention of pancreatic duct, failed ERCP, disconnected pancreatic tail syndrome (DPTS), stenosis of anastomoses after pancreas operation, pancreas divisum, chronic pancreatitis

## Abstract

**Background/Objectives**: Drawing upon over twenty years of clinical experience in endoscopic and endosonographic procedures, along with comprehensive literature research, we present an overview on EUS-guided pancreatography and pancreatic duct drainage (EUS-PD) as an alternative approach, encompassing indications, procedural methods, and outcomes, including complications and the success rate. **Methods:** Narrative review. **Results:** (corner points): EUS-PD is indicated for cases, for which conventional methods are ineffective due to altered abdominal anatomy of the upper gastrointestinal (GI) tract, such as congenital or postoperative conditions that prevent access to the papilla or pancreatoenteric anastomosis. It is also considered if there is symptomatic retention of the pancreatic duct due to pathological changes in the papillary region or stenosis of the pancreatic duct or anastomosis, especially if surgery is not feasible or poses higher risks. EUS-PD has a technical success rate ranging from 25 to 92%, albeit with a complication rate spanning from 14 to 40%, primarily comprising bleeding, perforation, pancreatitis, and pain. Long-term clinical success, measured by pain and symptom relief, falls within a range of 65–85%. **Conclusions:** The method offers advantages such as minimal invasiveness, enhanced quality of life, the potential for endoscopic revision in the case of complications, and compatibility with most conventional endoscopic instruments requiring extensive expertise in interventional endoscopy and endosonography.

## 1. Introduction

Endoscopic retrograde pancreatography (ERP) with drainage is widely considered the gold standard for the initial treatment of conditions marked by symptomatic pancreatic duct retention and dilatation (e.g., pancreas divisum, chronic pancreatitis, retention cysts, duct rupture following acute pancreatitis). Moreover, ERP plays a crucial role in managing complications following pancreatic surgery, including pancreatic fistulas, anastomotic stenosis, and cysts [1,2,3,4].

Several congenital, post-inflammatory, and postoperative changes may render the papilla of Vater (papilla) or anastomosis inaccessible or non-probable post-surgery. This condition affects approximately 10–15% of patients experiencing recurrent abdominal pain and pancreatic duct retention. Thus far, the only alternative for these patients has been enteroscopy-assisted endoscopic retrograde pancreatography (EA-ERP), which, however, demonstrates a low success rate ranging from 15 to 40%. As a result, surgery remains the final option for cases in which EA-ERP fails [5,6,7].

For patients with prior surgery and anastomotic stenosis or those experiencing disconnected pancreatic tail syndrome (DPTS) post-surgery or due to pancreatitis, traditional management often involved reoperation, carrying inherent risks and the potential need for pancreatectomy. However, endoscopic ultrasound (EUS), combining endoscopy with high-resolution transmural sonography, has evolved beyond its conventional diagnostic role to become pivotal in interventional therapy. Recent advancements have significantly broadened the scope of interventional endosonographic techniques. Notably, EUS has enabled the avoidance of surgical interventions in many cases, thus enhancing the safety of high-risk procedures [8,9,10,11,12,13,14,15,16].

Basically, endoscopic ultrasonography (EUS)-guided interventions are an essential tool of complex (such as combined or subsequent) therapeutic measures in current as well as future endoscopy, and an indispensable part of modern gastroenterology. Longitudinal EUS scanners allow for puncturing both mediastinal and abdominal lesions transluminally, which cannot be approached with other techniques. Using the EUS-guided puncture of such pathological lesions, it becomes possible to further perform more advanced endoscopic interventions, which, thus, become safer as well as more efficient, and they are associated with a lower complication rate compared with conventional endoscopic or even surgical interventions. A crucial aspect in interventional EUS is the adequate, less-traumatic treatment of pancreatic pseudocysts. The transluminal route of EUS-guided interventions spanning from the approach to placement of a drainage for abscesses and/or necroses is considerably easier by EUS guidance, including a better outcome.

Novel approaches and interventions include the internal EUS-guided insertion (from the upper GI tract) of the following:

(i) Transluminal cholangiodrainage, in patients with a malignant obstruction of the bile duct but no option to achieve sufficient conventional cholangiodrainage with ERC or PTC;
(ii) Transluminal pancreaticodrainage, in symptomatic patients with an enlarged pancreatic duct −/+ pancreatic fistula postoperatively or in patients with chronic pancreatitis,

which may be considered new therapeutic strategies with non-operative intention and/or low invasiveness.

In addition, for patients with malignant duodenal or gastric outlet stenosis, in particular, in the case of metastatic diseases, EUS-guided gastrojejunostomy, a safe bypass of malignant stenosis with a functionally sufficient passage, can be considered a reasonable alternative approach and be preferred to surgical procedures when conventional endoscopic interventions such as endoscopic (luminal enteral stents) and surgical (gastroenteroanastomosis) procedures fail or do not show a great prospect.

The aim of this concise review was to provide an overview of the emerging interventional endoscopic technique known as EUS-guided transluminal pancreatography and drainage of the pancreatic duct (EUS-PD), incorporating insights from existing literature alongside clinical and procedural experiences. Specifically, our objectives include the following:

- Outlining the indications for EUS-PD (such as symptomatic pancreatic duct retention with dilatation and failed ERP);
- Discussing the prerequisites for its implementation (including the need for advanced expertise in interventional endoscopy and interdisciplinary collaboration in visceral medicine);
- Assessing its feasibility and success rates (both technical and clinical, as well as the spectrum of associated complications).

Additionally, we aimed to carry out the following:

- Compare the method’s advantages and disadvantages;
- Critically evaluate its relevance for routine clinical practice.

## 2. Method

The narrative review is based on the following:

(i) Selective references from the current and topic-related scientific medical literature:
  - Possibly not older than 5 years;
  - Using keywords for the search such as “EUS-guided pancreatic duct drainage (EUS-PD[D])”, “Retention of pancreatic duct”, “Failed ERCP”, “Disconnected pancreatic tail syndrome (DPTS)”, “Stenosis of anastomoses after pancreas operation”, “Pancreas divisum”, “Chronic pancreatitis”.
(ii) Clinical experiences obtained from the daily management of affected patients who had undergone PD in the reporting department of interventional endoscopy and ultrasonography.

## 3. Results (Corner Points)

### 3.1. Indication

The clinical scenarios characterized by pancreatic duct retention with dilatation and recurrent pancreatitis or chronic pain where ERP, the gold standard for ductal relief, proves ineffective, encompass several key aspects:

- Endoscopic inaccessibility of the papilla/anastomosis: Occurs post-BII gastric resection, post-[sub-]total gastrectomy, post-pylorus-preserving pancreatoduodenectomy (PPPD) (each with Roux-en-Y reconstruction of the upper GI tract anatomy), and post Kausch–Whipple operation.
- Non-probing of the pancreatic duct via minor or major papilla due to pathological papillary changes: Seen in chronic pancreatitis, papillary sclerosis, or the anatomical variant pancreas divisum.

Fundamentally, the clinical indication profile aligns with that of ERP. In cases of impaired pancreatic duct drainage leading to elevated intraductal pressure and associated clinical pain symptoms, the objective was to optimize drainage by inserting a drain.

Primary pathological indications encompass the following:

- Symptomatic pancreas divisum;
- Chronic pancreatitis;
- Pancreaticocutaneous fistula (following pancreatic pseudocyst/abscess drainage without sufficient spontaneous duct drainage, after pancreatic left or tail resection with fistula at the resection site, and after endoscopic transgastric necrosectomies);
- DPTS subsequent to necrotizing pancreatitis with gangrene or after previous open surgical or endoscopic necrosectomy;
- Symptomatic stenosis following pancreatojejunostomy.

### 3.2. Principle

When the papilla cannot be accessed or probed, an alternative route to the pancreatic duct is required. This necessitates real-time imaging capable of identifying relevant anatomical structures. Integrating EUS and fluoroscopy, potentially with ERP as a rendezvous technique, proves optimal. The preferred approach involves transluminal access, typically transgastrically, to ensure the shortest distance and avoid vascular structures.

### 3.3. Technique

**Figure 1:**
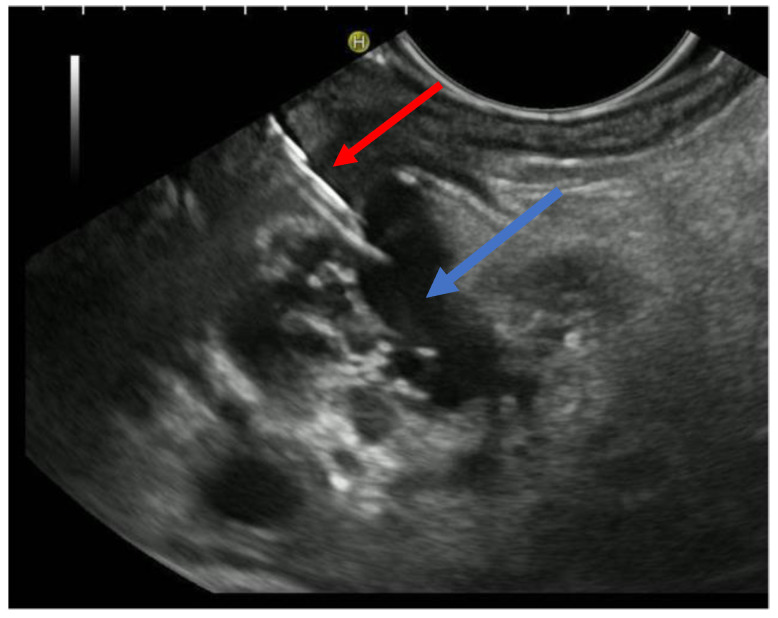
Intraprocedural EUS image: Patients with remitting pancreatitis and dilated pancreatic duct; transgastric EUS-FNA (red arrow) of the dilated duct (blue arrows) and pancreatography (Figure 2), selected from the clinical picture library of the reporting Dept. of Gastroenterology, Hepatology and General Internal Medicine.

**Figure 2:**
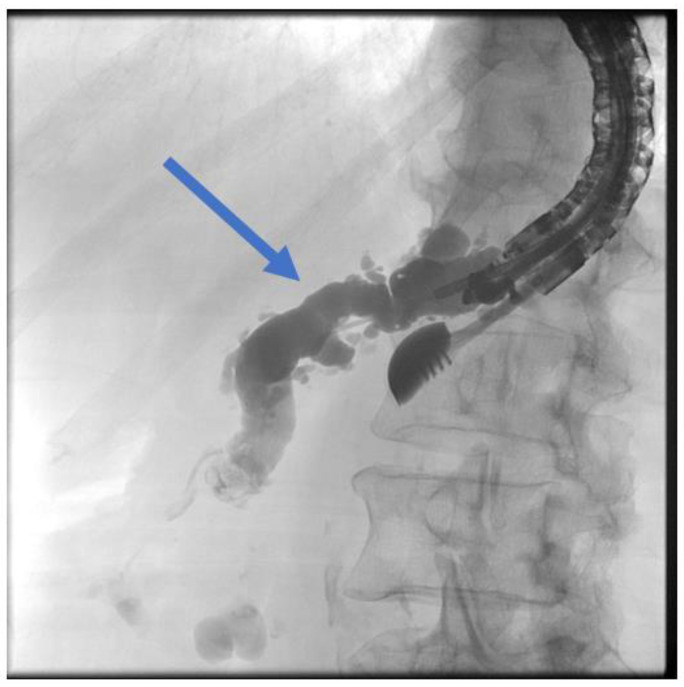
Intraprocedural fluoroscopy (during EUS-guided procedure): Legend see also Figure 1.

The patient is positioned prone on an X-ray fluoroscopy table and undergoes endoscopic ultrasound (EUS) using a therapeutic longitudinal scanner (e.g., EG 3270 UK, Hitachi Ultraschall, Berlin, Germany; GF-UCT 180, Olympus, Hamburg, Germany; Fuji EG-580UT2, Tokyo, Japan) following unsuccessful conventional ERP, with peri-interventional antibiotic administration i. v. (2 g ceftriaxone or 4 g tazobactam). Once the pancreatic duct is identified, a 19-G needle (Boston Scientific, Ratingen, Germany; Olympus, Hamburg, Germany) is used to puncture it in the direction of the anastomosis or papilla. Pancreatic juice is aspirated and sent for microbiological and cytological examination. Subsequently, the pancreatic duct is visualized via X-ray fluoroscopy following the instillation of the contrast medium (Figure 1 and Figure 2—selected from the clinical picture library of the reporting Dept. of Gastroenterology, Hepatology and General Internal Medicine as all the following figures).

**Figure 3:**
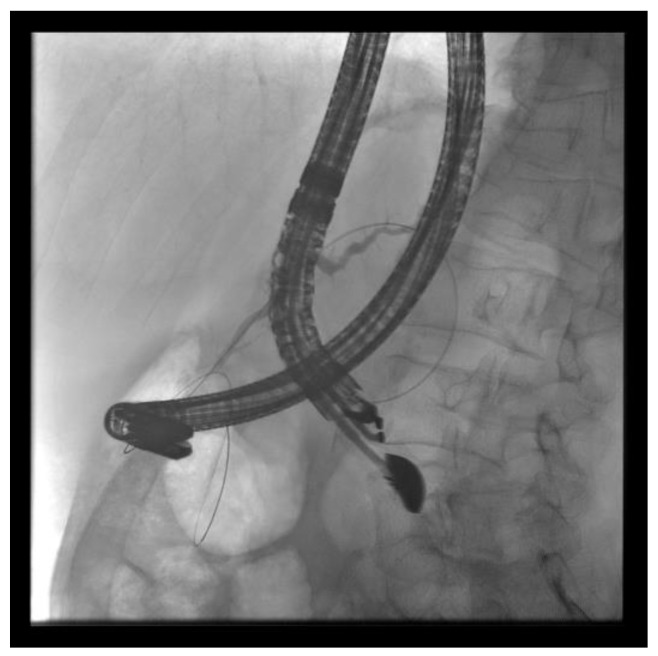
EUS puncture with a 19-G needle tangentially toward the papilla; the wire is passed out of the papilla via a ring knife, followed by device change of the duodenoscope.

**Figure 4:**
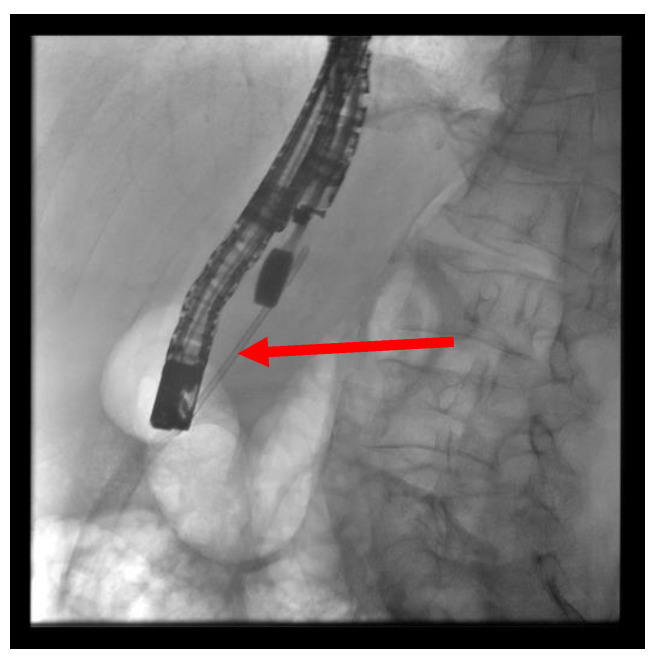
After the device change, the wire is grasped with forceps and passed out, followed by a conventional ERP with stent placement (red arrow).

**Figure 5:**
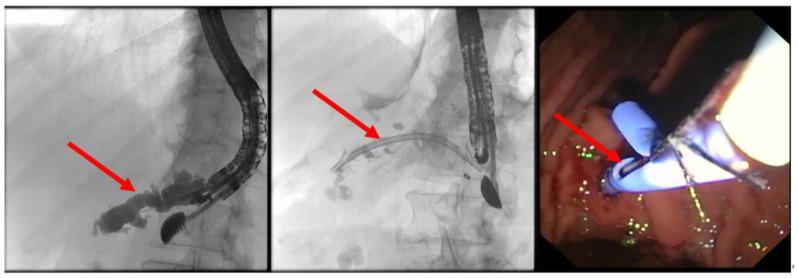
Dilated pancreatic duct (red arrow) in history of necrotizing pancreatitis—suspected DPTS; EUS-guided pancreatography (**left panel**) with following insertion of a plastic prosthesis (red arrow) for retrograde drainage (**middle panel**) and endoluminal (endoscopic) control of the right plastic prosthesis position (red arrow—**right panel**); selected from the clinical picture library of the reporting Dept. of Gastroenterology, Hepatology and General Internal Medicine.

A 0.035-inch guidewire (Boston Scientific, Ratingen, Germany) is inserted and, if possible, advanced to the papilla or anastomosis without manipulating the needle to prevent wire shearing. If the wire initially fails to pass through the papilla or anastomosis, it can be manipulated using specialized wires after access with the ring knife (MTW Endoskopie Manufaktur W. Haag KG, Wesel, Germany) to prevent the risk of wire shearing.

(A) EUS-ERP rendezvous technique: If the wire can be passed through the papilla and the papilla is accessible with the endoscope, a duodenoscope is employed. The guidewire is then grasped with forceps and pulled out, enabling subsequent conventional ERP with endoscopic papillary balloon dilation (EPBD) and stent placement (Figure 3 and Figure 4).

(B) EUS-guided pancreatic duct drainage (EUS-PD): If the wire cannot pass through the papilla or if the papilla is unreachable with a standard duodenoscope or enteroscope (e.g., post-gastrectomy, Roux-en-Y reconstruction, Kausch–Whipple surgery; see the above), the wire is advanced as far as possible into the pancreatic duct following an EUS puncture. An access site is then created along the wire using a Will HF ring knife (MTW Endoskopie Manufaktur W. Haag KG, Wesel, Germany) followed by dilatation of the typically transgastric access using a 6-mm bile duct dilatation balloon (Boston Scientific, Ratingen, Germany). The choice of prosthesis, which is inserted, depends on factors such as the site and width of the pancreatic duct system or the direction of the puncture. If a wide pancreatic duct is punctured near the obstruction, antegrade drainage (pancreaticogastrostomy/pancreaticoduodenectomy) can be attempted by using a self-expanding metal stent (SEM or LAMS, Boston Scientific, Ratingen, Germany) or a straight plastic Amsterdam prosthesis (Medi-Globe, Achenmühle, Germany) (Figure 5).

**Figure 6:**
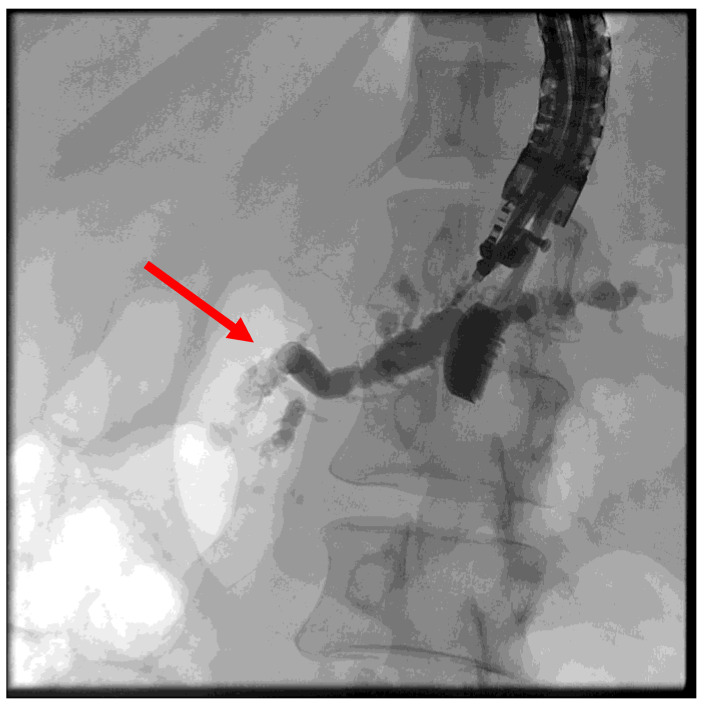
Puncture of the pancreatic duct in the tail segment with unsuccessful wire advancement due to stricture (red arrow).

**Figure 7:**
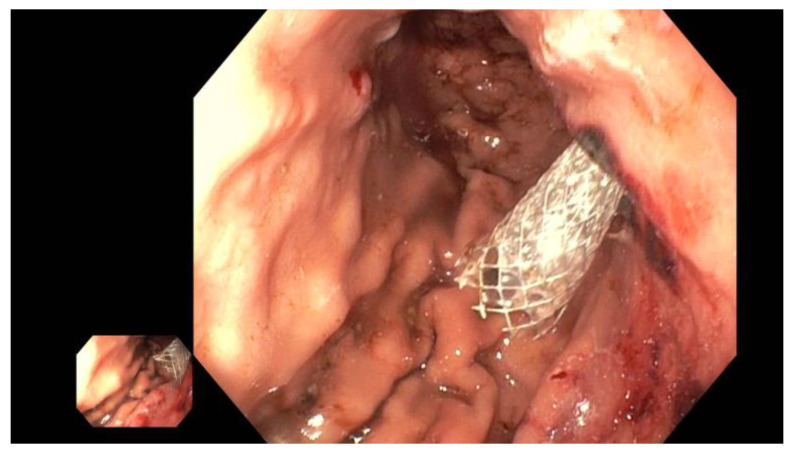
Placement of a self-expanding metal stent (red arrow) for pancreaticogastrostomy.

In cases where the puncture is distant from the obstruction (such as in the pancreatic body or tail), retrograde drainage into the stomach (pancreaticogastrostomy) is pursued. This procedure also utilizes metal stents or straight plastic stents (Figure 6 and Figure 7).

Metal stents, with their wider lumen width (10 mm), are best utilized if an obstruction of secondary ducts or significant portions of the main duct can be ruled out. For central pancreatic duct punctures, plastic stents are often preferred as they allow for the transport of pancreatic juice from both the head and tail segments, given that they typically do not completely occlude the lumen.

**Figure 8:**
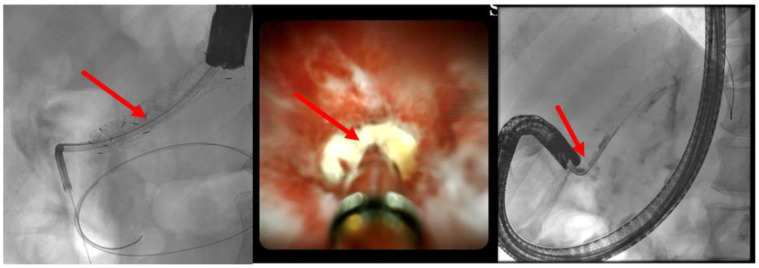
Following the retrograde placement of a self-expanding metal stent (red arrow)—left panel, the obstructing pancreaticolith is destroyed with an EHL probe (red arrow; middle panel), restoring the pathway through the papilla (red arrow; fluoroscopy control—right panel), selected from the clinical picture library of the reporting Dept. of Gastroenterology, Hepatology and General Internal Medicine.

**Figure 9:**
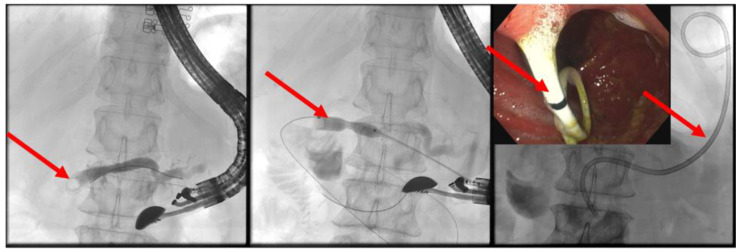
Post-pancreatojejunostomy resection—remitting pain—dilated pancreatic duct; transgastric puncture of the pancreatic duct—demonstration of a pancreaticolith (red arrow, to the anastomotic stenosis (left panel); balloon dilation (red arrow) of the stenosis, advancement of the stone in push technique (middle panel); placement of jejuno-pancreatico-gastrostomy drainage (red arrows; 8.5-Fr. double pigtail); right panel (satellite panel, endoscopic control view), selected from the clinical picture library of the reporting Dept. of Gastroenterology, Hepatology and General Internal Medicine.

The advantage of using a metal stent (such as Hanaro^®^ by Olympus, Hamburg, Germany, or SEM by Boston, Ratingen, Germany) lies in the possibility of later transmural targeted intervention in the pancreatic duct with a pancreaticoscope (such as SpyGlass by Boston Scientific, Ratingen, Germany). This option should be considered if primarily impassable strictures or obstructing pancreaticoliths are suspected, as these can be treated via targeted visual inspection. Pancreaticoliths, for example, can be fragmented and removed using pancreaticoscopic electrohydraulic lithotripsy (EHL), thereby restoring physiological antegrade drainage (Figure 8).

(C) EUS-guided internal antegrade pancreatic duct drainage: In cases where the papilla or anastomosis cannot be accessed, a wire is inserted following the puncture of the pancreatic duct. Subsequently, an HF ring knife (MTW Endoskopie Manufaktur W. Haag KG, Wesel, Germany) is introduced via the wire, which can also be employed to overcome challenging strictures with specialized wires, as there is no risk of wire ablation. If the stricture at the anastomosis can be passed, an 8- to 15-mm dilatation balloon is inserted to dilate the stenosis (refer to Figure 9). Additionally, a long pigtail (14 cm, 8.5 French) can be inserted as jejuno-pancreatico-gastrostomy (ring drainage) to secure the dilatation effect, which can be removed after approximately 8–12 weeks.

**Figure 10:**
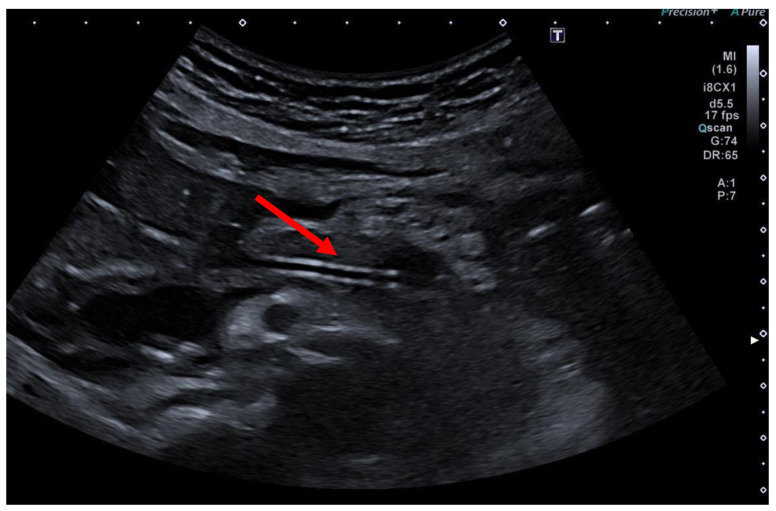
Postinterventional ultrasound control after EUS-PD revealing a decompressed pancreatic duct with a transgastric plastic stent in place (red arrow), selected from the clinical picture library of the reporting Dept. of Gastroenterology, Hepatology and General Internal Medicine.

A transabdominal ultrasound examination on the first post-interventional day is essential to monitor successful treatment. A decrease in the diameter of the pancreatic duct compared to previous observations and the detection of air within the duct are signs of an effective drainage (Figure 10).

The placement of the drainage within both the pancreatic duct and intestinal lumen should be meticulously documented. Pancreatic duct stents inserted transgastrically pose a lower risk of displacement compared to bile duct stents. Furthermore, vigilance is required to monitor fluid accumulation in the lesser sac, potentially indicating therapy-induced pancreatic duct leakage or stent malfunction.

Technical success is affirmed by adequate pancreatography post-pancreatic duct puncture and successful stent deployment, leading to a reduced pancreatic duct diameter with the presence of air within the duct lumen.

Clinical success, indicating sufficient internal drainage, is characterized by pain alleviation or the cessation of pancreaticocutaneous fistula drainage.

### 3.4. Complications

Aside from common, but typically mild, post-interventional pain, effectively managed with analgesics, potential complications encompass bleeding, perforation, and stent migration or occlusion. To prevent migration, it is advisable to maintain a short distance between the intestinal lumen and pancreatic duct. Stent migration can be effectively thwarted by securing the visible end of the stent intraluminally with clips on the upper GI tract wall or with stent barbs directly. Should a complication necessitate surgical revision, the imperative of preserving minimal invasiveness is compromised. Therefore, intervention should be performed at a specialized abdominal surgery center with high expertise to effectively address the resulting increase in invasiveness.

### 3.5. Advantages/Disadvantages

The primary advantage of EUS-PD lies in its unique access route to the pancreatic duct, which cannot be achieved with other interventional procedures. This approach is particularly beneficial for patients with recent pancreatic surgery and associated complications such as anastomotic stenosis or chronic pancreatic fistula, aiming to avoid secondary surgery. The procedure’s minimal invasiveness and adaptability enable effective internal pancreatic duct drainage, leading to an improved quality of life. Moreover, the option for endoscopic revision in the case of complications enhances its appeal. Following the use of metal stents, therapy can be extended through transluminal interventions in the pancreatic duct with the assistance of pancreaticoscopy. The endoscopic instruments utilized (including endosonography devices, longitudinal scanners, duodenoscopes, X-ray fluoroscopy devices, puncture needles, guide wires, dilatation balloons, ring knifes, and plastic/metal prostheses) are comparable to those used for internal EUS-guided cyst drainage. However, the main drawback lies in the exceptionally high level of interventional endoscopic/-sonographic expertise required for EUS-PD application. Additionally, challenges include the relatively low case volume, long learning curve, and clinical success rates averaging approximately 70–85%, which still have room for improvement.

### 3.6. Follow-Up Care

The objective of clinical and imaging follow-up is to exclude or detect complications such as intervention-related bleeding, perforation, and stent migration, typically presenting as post-interventional pain. Ultrasound is generally effective in documenting the correct stent position; however, if ultrasound conditions are inadequate, a CT scan should be considered. Special attention should be given to new fluid collections in the omental bursa and peri-intestinally in the lower abdomen. The puncture of fluid with lipase determination can aid in the early detection of a faulty drainage system.

Follow-up evaluations recommended every 6–12 months should encompass clinical examination, transabdominal ultrasound, and laboratory tests (including CRP, white blood cell count, and lipase). Symptoms reminiscent of those prior to intervention should be carefully monitored. If clinical symptoms improve and the pancreatic duct widens without evidence of air, drainage malfunction or obstruction should be suspected, prompting consideration for reintervention. An elective replacement of prostheses was not favored in the own patient population; however, targeted reintervention was performed in cases of recurrent symptoms.

## 4. Discussion

The main cause of pain in patients diagnosed with an enlarged pancreatic duct, in whom a malignant tumor has been ruled out, is ductal hypertension resulting from obstruction [17]. The underlying causes of pancreatic duct retention with dilatation include chronic pancreatitis with ductal system stenosis, obstructions due to calcifications and pancreatic calculi, as well as ductal ruptures. Furthermore, inflammatory papillary stenoses, anatomical variations (such as pancreas divisum), and stenoses following surgeries contribute to pancreatic duct retention with dilatation [18]. The objective of pain management in these patients is to alleviate congestion in the pancreatic duct either through surgical or endoscopic interventions. Success rates ranging from 70 to 90% have been demonstrated in both endoscopic and surgical studies [18,19,20,21,22,23,24,25]. The endoscopic transpapillary drainage of the congested pancreatic duct reaches its limits in the presence of anatomical variants (e.g., pancreas divisum) or following surgeries (e.g., B-II gastric resection, Roux-en-Y anastomosis). Treating obstructive pancreatitis in the remnant pancreas after pancreatic head resections due to stenoses at the anastomosis or chronic pancreatic fistulas following pancreatic surgeries presents a significant challenge. Often, a repeat surgery becomes the last resort to improve the patient’s condition. 

EUS-PD emerges as a viable alternative for achieving the drainage of a dilated pancreatic duct system in patients with pancreatic duct obstruction and unsuccessful transpapillary drainage [18,26,27,28,29,30,31,32,33,34,35,36] (Table 1). All necessary diagnostic assessments to rule out malignant pancreatic conditions should be conducted prior to EUS-PD. In cases of uncertainty, an exploratory laparotomy with a duodenum-preserving pancreatic head resection should always be considered in symptomatic chronic pancreatitis [21]. In a series of transintestinal EUS-guided pancreatic duct drainages, malignancy was detected in 5 out of 36 patients. Additionally, in a study by Ergun, pancreatic carcinoma was diagnosed in 3 out of 20 cases (15%) following EUS-PD [18,37]. In our series of 207 patients, 5 individuals subsequently developed pancreatic cancer. Among the 24 surgically treated patients with chronic pancreatitis, pancreatic carcinoma was detected in 3 cases (12.5%). This highlights the importance of meticulous patient selection for EUS-PD, particularly in those with an intact pancreas and chronic pancreatitis. The choice for exploratory laparotomy warrants careful consideration in cases of chronic pancreatitis accompanied by pancreatic duct retention with dilatation and clinical symptoms. EUS-PD should be contemplated only if contraindications, such as portal hypertension or liver cirrhosis, are absent, or if the patient vehemently opposes surgery.

**Figure 11:**
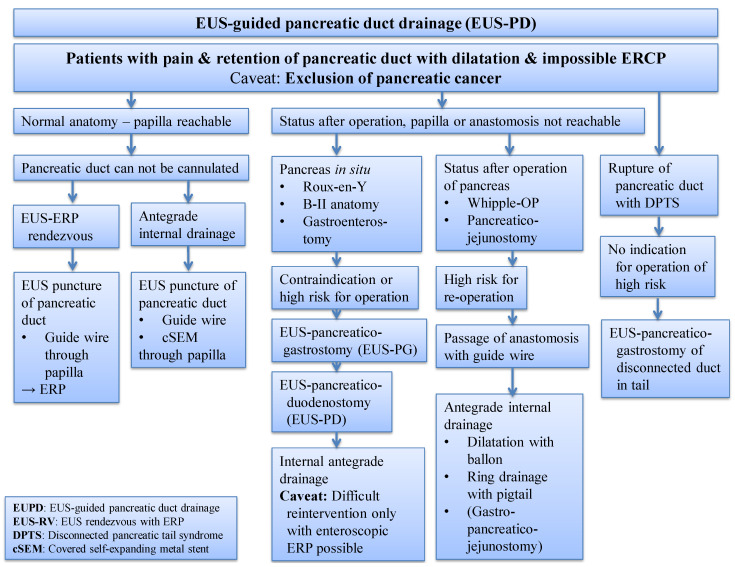
Rational procedure for symptomatic retention with dilatation of the pancreatic duct; EUS-PD: EUS-guided pancreatography and pancreatic duct drainage; EUS-RV: EUS rendezvous procedure with ERP; DPTS: disconnected pancreatic tail syndrome; selected from the clinical picture library of the reporting Dept. of Gastroenterology, Hepatology and General Internal Medicine.

Essentially, three strategies for conducting EUS-PD are outlined, as shown in Figure 11, depending on the indication:

1. If the papilla is accessible but pancreatic duct probing is unsuccessful or stenosis persists, a rendezvous procedure is initially preferred;
2. In cases where the papilla cannot be reached, primary transintestinal EUS drainage is utilized, allowing for either antegrade or retrograde pancreatic secretion drainage.

Alternatively, transintestinal intervention at the anastomosis, involving balloon dilatation with temporary insertion of a ring drain, offers a solution for addressing complications following pancreatojejunostomy.

While the puncture of the pancreatic duct and pancreaticography achieve success rates in nearly 100% of the cases, the insertion of a drain may not always be feasible. Technical success rates ranging from 25 to 91% have been reported in the literature case series (Table 1). Various techniques for pancreaticoenterostomy placement are described in these case series, including the use of a diathermia knife, bougies, balloons, and stent retrievers. Comparing the results across studies is difficult due to heterogeneous patient populations and small sample sizes. The reported complications range from 14 to 25%, including bleeding, pseudocyst formation, perforation, and pancreatitis, with no method-related mortality observed in any of the case series. Long-term clinical success rates range from 69 to 80%, comparable to results from transpapillary and surgical drainage procedures [21,24,25,38]. Stent migrations and obstructions are noted as long-term issues in 20 to 55% of cases, necessitating reinterventions [18].

Drawing from current personal experience and the literature data, EUS-PD emerges as a safe alternative for draining dilated pancreatic duct systems in symptomatic patients who cannot be accessed via conventional endoscopic methods (Table 1).

Particularly suitable candidates for EUS-PD include high-risk patients such as those with liver cirrhosis or portal hypertension, as well as individuals who have undergone surgery and exhibit persisting pancreatic fistulas or anastomotic stenoses resulting in pancreatic duct retention with dilatation. Currently, EUS-PD should only be performed by experienced interventional endosonographers at large gastroenterology centers with multidisciplinary expertise, including abdominal surgeons and interventional radiologists, especially for managing complications.

According to the 2022 ESGE guideline, EUS-PD is recommended for symptomatic patients with pancreatic duct retention if drainage is unfeasible with ERP or ER-ERP. The procedure should be reserved for experts at specialized centers. Rendezvous procedures are preferred over transgastric drainage when the papilla is accessible [39,40].

**Table 1:** EUS-PD technical and clinical success incl. adverse event rates reported in the literature (chronological order; “N. D.”, not determined).

Ref.	Year	Country	[*n*]	Technical Success (%)	Clinical Success (%)	Adverse Events (%)
[26]	2004	US	4	25	N. D.	25
[27]	2007	Germany	14	69	78	15
[28]	2007	US	13	76	69	15
[18]	2007	France/Belgium	36	92	69	14
[41]	2009	US	8	88	50	0
[42]	2010	US	21	48	80	10
[37]	2011	Belgium	20	90	72	19
[10]	2011	Germany	65	60	74	15
[43]	2012	US	25	88	N. D.	16
[44]	2012	Spain	19	58	N. D.	26
[45]	2013	Japan	14	88	N. D.	6
[46]	2013	US	45	74	83	6
[47]	2014	Germany	84	59	78	18
[48]	2016	Korea	25	100	100	17
[29]	2017	US, Brazil, France	80	89	91	20
[38]	2017	US, Belgium, Japan, Brazil	40	93	94	41
[34]	2018	Japan	30	100	98	23
[49]	2018	Japan	15	87	92	27
[32]	2019	India	44	88	81	22
[50]	2020	Korea	23	100	100	20
[51]	2020	Meta-analysis (22 St.)	714	77	89	18
[30]	2020	Meta-analysis (9 St.)	401	85	88	25
[33]	2021	Meta-analysis (16. St.)	503	81	84	21
[31]	2022	Japan	45	91	97	30
Own study	2024	Germany	207	75	83	17

The next important aspect is potential adverse reactions such as pancreatitis—compared to the use of ERCP—as found in the publication by Boicean et al., 2023 [52]. In this context, ERCP is the gold standard for treating pancreatic or biliary duct obstructions. Potential complications include bleeding, infections, perforations, and acute pancreatitis. If the pancreatic or biliary duct ostium is not endoscopically accessible or cannulable, EUS-guided drainage presents a minimally invasive alternative to surgery. Similar to ERCP, EUS-PD carries risks such as bleeding, paravasation, abscess or pseudocyst formation, and procedure-induced acute pancreatitis.

This paper addresses—in particular—pancreatic duct obstructions. In cases of pancreaticolithiasis with sufficient access, typically provided by a metal stent, pancreatic stone extraction can also be performed via endoscopic ultrasound-guided drainage.

### 4.1. Basic Considerations Related to EUS-PD

- EUS can be considered an important skill since it can clarify differential diagnosis, as impressively shown by Boicean et al. [53] In particular, the article reveals the suitable role of ultrasound and echo-endoscopy; in this review, the diagnostic significance of ultrasonography and endoscopic ultrasound (EUS) in accurately identifying various GI lesions were underscored. The pivotal role of EUS and abdominal ultrasound in achieving an accurate diagnosis finding was emphasized, and awareness among clinicians with regard to the importance of multimodal imaging approaches for optimal patient management was raised.
- According to Ciu et Kozarek (2023) [54], EUS—in addition to therapeutic ERCP (the workhorse for biliary and pancreatic ductal interventions [40])—(i) is an “important tool for imaging complications of pancreatitis and has (helped to) significantly reduce(d) the need for surgery”, (ii) can be helpful in “managing pancreatic duct leaks” in more complex cases as part of “a multidisciplinary approach with interventional radiology”, and (iii) can guide “antitumor therapy”, which “remains investigational, although (it) shows potential in palliation and cure of pancreatic neoplasms”.
- Vitali et al. [55] favor EUS as “a fundamental tool for diagnosis and therapeutic procedures in gastroenterology, hepatology, and pancreatology; it combines the profits of endoscopy to gain access to human cavities with the features of high-frequency ultrasound probes to achieve superior visualization of anatomical regions, making pinpoint diagnostics possible”. “EUS also enables the following:
  * Interventional direct access to the pancreatic parenchyma and the retroperitoneal space, the pancreatic duct, the pancreatic masses, cysts, and vascular structures for diagnostic and therapeutic purposes;
  * Ways to perform pseudocyst drainage, necrosectomy, transenteral drainage, and transenteric access of the main pancreatic duct for the direct visualization or therapy of vascular structures adjacent to the pancreas”.

“EUS has deep impact on pancreatology and the development … has increased in the last years exponentially”.

- Most of published data related to EUS-PD are retrospective, involving case reports and case series [40].

### 4.2. Specific Technical Aspects

- Using two endoscopes (as one additional resource) simultaneously:

In exceptional cases, we use two endoscopes to reduce the risk of wire dislocation, particularly when the wire position is highly unstable. The image illustrates the rendezvous technique where the wire is advanced over the papilla and retrieved with an ERCP device to facilitate the placement of an anterograde pancreatic duct drain. However, in recent years, we have increasingly moved away from this approach, where feasible, in favor of placing an internal antegrade drain. This method involves advancing the prosthesis through the endosonographically created access to a position above the papilla, allowing for pancreatic secretions to drain into the small intestine.

- Can the guidewire passed into (and through) the pancreatic duct and into the gut simply be secured by one or more endoscope clips (in contrast to the risks and resource utilization of passing simultaneous or sequential endoscopes per os)?
- A few endoscopists suggest to use a 22-gauge needle and 0.018-inch guidewire for non-dilated PD access, including the fact that there is less shearing of a 0.025-inch guidewire through a 19-Fr. needle, which appears to be an interesting aspect. At our institution, we exclusively use a 19-gauge needle with a 0.035-inch wire to puncture the pancreatic duct. This approach yields a nearly 100% technical success rate for pancreatic duct puncture and subsequent pancreatography, even in cases with accentuated or mildly dilated ducts. If the pancreatic duct is not dilated, the puncture is not indicated.

We, in particular, have no experience with shearing when using a 0.025-inch guidewire with a 19-gauge needle, as we do not employ this combination. However, the suggestion is understandable.

To minimize the risk of shearing, the wire should be manipulated as little as possible over the needle. The risk is further reduced if the needle is slightly retracted into the endoscope’s working channel or if the wire is manipulated over the ring knife.

- From a very practical point of view, the vast majority of institutions routinely image patients after a EUS-PD utilizing CT. This might be due to usual rather limited expertise in wide-spread use of transabdominal ultrasound for various purposes and a lack of advanced sonographers. However, as the authors stated, a transabdominal ultrasound is the preferred diagnostic tool of choice for post-procedure imaging, easily revealing “a decompressed pancreatic duct with transgastric plastic stent in place (red arrow)” as expressed in the legend of Figure 10.
- It might be amazing that image-guided radiology does not play a predominant role, e. g., in pancreatic necrosis, and instead of that, endoscopic or surgical necrosectomy only. The “philosophy” of the reporting department is that such image-guided interventions, e. g., for external drainage, preferentially at an initial stage of pancreatitis, can also be easily executed using ultrasound for image guidance in experienced hands. Therefore, image-guided radiology is only needed in very limited indications in daily clinical practice of the reporting department.
- Considering the paucity of discussion that DPDA with an external pancreatic fistula is more often the consequence of percutaneous drainage than of surgical or endoscopic drainage of walled-off pancreatic necrosis (WOPN), it can be stated that a possible complication of percutaneous drainage, particularly when used alone for WOPN, is a persistent percutaneous pancreatic fistula [56,57]. Ideally, this complication should resolve with optimized drainage of pancreatic secretions from the duct. Gupta et al. report a complication rate (including bleeding, fistulas, and infections) of 21.4% for percutaneous drainage [58]. Compared to the endoscopic or surgical drainage of WOPN, the risk of developing a percutaneous pancreatic fistula is significantly higher, and the success rate of standalone percutaneous drainage is considerably lower. Combining percutaneous and endoscopic drainage techniques significantly reduces the complication rate for percutaneous fistulas, as this approach usually ensures more complete and lasting drainage. Several studies have shown that endoscopic drainage techniques have a lower rate of pancreatic fistulas compared to surgical drainage [57,59,60,61,62].
- A further usual comment or critique is that pancreatic duct stents occlude, migrate, erode, fracture, etc., with variable consequences, some of which occur later on, at a time distant from the procedure. In contrast, a patient stented for benign biliary disease would not be left uncontrolled, e. g., with no regular follow-up controls. What about “following-up” the EUS-PD patients regularly, including reimaging, and appropriately managing stent dysfunction, as well as the case of developing exocrine and endocrine insufficiency, and the subsequent development of pancreatic cancer?

Though it needs to be taken into account, based on the great case-related clinical experience obtained in treating such patients with EUS-PD over the time period, the described approach, including follow-up management recommendations, proved excellently in practice and can be advised.

### 4.3. Final Assessment of EUS-PD

Giovanni [63] concluded that “considering the major limitations in alternative treatment options after failed ERCP, EUS-guided pancreatic duct drainage has the potential to become standard-of-care by avoiding more invasive and involved surgical interventions”, “is a valuable skill in the interventional endoscopist’s armamentarium” [36] and “serves as a rescue procedure” [36], however, with only a low frequency “ranging between two and four cases per centre per year” [36]. In addition, “though data has demonstrated that the procedure can be safe and effective, EUS-guided PD drainage remains one of the most technically challenging therapeutic EUS interventions, as evidenced by the multiple considerations on device selection (e.g., small caliber accessories) and the risk of severe complications” [63] (with only a low level of satisfying standardization [36,40]). According to training and learning–curve aspects raised by Teh et al. [36], procedural efficiency was achieved after 27 procedures and proficiency after approximately 40 procedures, respectively. Interestingly, considerations (and even guidelines) on the procedure are predominated by guiding statements due to “low-quality evidence” than by reliable data obtained in “larger prospective and long-term comparative studies (e.g., direct head-to-head comparisons) in the field of EUS-PDD”, which need to be consequently divided into the outcomes of EUS-PD as rendezvous manoeuvre (ring drainage) vs. “transmural stenting” [36].

### 4.4. Possible (Still-Existing) Limitations and Aspects in Discussion

Open questions regarding the use of EUS-PD include the following:

1. Is EUS-PD suitable for patients with chronic pancreatitis despite the 5–10% incidence of carcinoma in this population? Suggestion: Reserve for high-risk patients (e.g., portal hypertension, liver cirrhosis) or those unwilling to undergo surgery.
2. Should EUS-PD primarily employ the rendezvous technique if the papilla is accessible and ERP fails, or is transintestinal drainage feasible for creating a neoostium or pancreatic fistula? Suggestion: Prefer rendezvous whenever feasible!
3. Which stent type is preferable for drainage: partially covered metal stent or plastic stent? Suggestion: Adapt stent selection to pancreatic duct width, ensuring no obstruction of the upstream duct. Consider 7-Fr. or 8.5-Fr. plastic prostheses after balloon dilatation to 6 mm. Metal stents allow for elective interventions like EHL in pancreaticolithiasis.
4. Should stent replacement be scheduled, or should reintervention be based on clinical symptoms? Suggestion: Reintervention after successful drainage only if symptoms recur; avoid elective stent changes!
5. Is temporary stenting necessary for the formation of a functional pancreatointestinal fistula, or is an HF fistulotomy sufficient? Suggestion: Fistulotomy and balloon dilatation with the intention of stenting should always be performed whenever possible. If stent placement fails, the possibility of a chronic fistula in the functional pancreas theoretically exists if fistulotomy and balloon dilatation have been performed.

## 5. Conclusions

EUS-PD is an elegant, low-risk (in experienced hands), successful and complementary method for ERP, for a strictly selected patient population to achieve endoscopic drainage of the pancreatic duct in cases of obstruction.

Serving as an alternative approach, it expands therapeutic options for interventional endoscopists effectively and with minimal invasiveness, potentially avoiding significantly more invasive surgical interventions.

Randomized studies similar to ERP vs. surgery in chronic pancreatitis will not be conducted with this procedure, so further results from larger prospective studies are needed to critically assess the indications, and technical and clinical success rates, as well as the complications and outcomes.

Currently, the method serves as a rescue therapy before surgery or reoperation in patients with symptomatic pancreatic duct retention with dilatation and frustrating ERP, and should only be applied at expert centers proficient in interventional endoscopy/sonography for an appropriate EUS-PD, and to ensure the safe management of complications, supported by expertise in abdominal surgery and interventional radiology.

## Data Availability

The original contributions presented in the study are included in the article, further inquiries can be directed to the corresponding author/s.

## List of Abbreviations

BII	Billroth-II
CT	Computed tomography
CRP	C-reactive protein
DPTS	Disconnected pancreatic tail syndrome
EHL	Electrohydraulic lithotripsy
ERP	Endoscopic retrograde pancreatography
EPBD	Endoscopic papillary balloon dilatation
EUS	Endoscopic ultrasonography
EA-ERP	Enteroscopy-assisted endoscopic retrograde pancreatography
EUS-FNA	EUS-guided fine-needle aspiration
EUS-PD	EUS-guided pancreatography and pancreatic duct drainage
ESGE	European Society of Gastrointestinal Endoscopy
Fig.	Figure
Fr.	French
G	Gauge
GI	Gastrointestinal
HF	High frequency
LAMS	Lumen-apposing metal stent
PPPD	Pylorus-preserving pancreatoduodenectomy
SEM	Self-expanding metal stent

## References

[1] Dumonceau J.-M., Delhaye M., Tringali A., Arvanitakis M., Sanchez-Yague A., Vaysse T., Aithal G.P., Anderloni A., Bruno M., Cantú P. (2019). Endoscopic treatment of chronic pancreatitis: European Society of Gastrointestinal Endoscopy (ESGE) Guideline—Updated August 2018. Endoscopy.

[2] Cotton P.B. (2012). Endoscopic retrograde cholangiopancreatography: Maximizing benefits and minimizing risks. Gastrointest. Endosc. Clin. N. Am..

[3] Freeman M.L. (2012). Complications of endoscopic retrograde cholangiopancreatography: Avoidance and management. Gastrointest. Endosc. Clin. N. Am..

[4] Hoffmeister A., Mayerle J., Beglinger C., Büchler M.W., Bufler P., Dathe K., Fölsch U.R., Friess H., Izbicki J., Chronic Pancreatitis German Society of Digestive and Metabolic Diseases (DGVS) (2012). [S3-Consensus guidelines on definition, etiology, diagnosis and medical, endoscopic and surgical management of chronic pancreatitis German Society of Digestive and Metabolic Diseases (DGVS)]. Z. Gastroenterol..

[5] Ali U.A., Pahlplatz J.M., Nealon W.H., Van Goor H., Gooszen H.G., Boermeester M.A. (2015). Endoscopic or surgical intervention for painful obstructive chronic pancreatitis. Cochrane Database Syst. Rev..

[6] Ayoub F., Brar T.S., Banerjee D., Abbas A.M., Wang Y., Yang D., Draganov P.V. (2020). Laparoscopy-assisted versus enteroscopy-assisted endoscopic retrograde cholangiopancreatography (ERCP) in Roux-en-Y gastric bypass: A meta-analysis. Endosc. Int. Open.

[7] Ross A.S. (2009). Endoscopic retrograde cholangiopancreatography in the surgically modified gastrointestinal tract. Gastrointest. Endosc. Clin. N. Am..

[8] Perez-Miranda M., Barclay R.L., Kahaleh M. (2012). Endoscopic ultrasonography-guided endoscopic retrograde cholangiopancreatography: Endosonographic cholangiopancreatography. Gastrointest. Endosc. Clin. N. Am..

[9] Will U., Meyer F. (2012). [Endoscopic ultrasonography (EUS)-guided transluminal cholangiodrainage (EUCD)—A novel option of interventional endoscopy in the interdiciplinary management of obstructive jaundice]. Zentralbl. Chir..

[10] Will U., Wanzar C., Gerlach R., Meyer F. (2011). Interventional ultrasound-guided procedures in pancreatic pseudocysts, abscesses and infected necroses—Treatment algorithm in a large single-center study. Ultraschall Med.-Eur. J. Ultrasound.

[11] Will U. [Therapeutic endosonography]. Z. Gastroenterol..

[12] Khashab M.A., Varadarajulu S. (2012). Endoscopic ultrasonography as a therapeutic modality. Curr. Opin. Gastroenterol..

[13] Kaul V., Adler D.G., Conway J.D., Farraye F.A., Kantsevoy S.V., Kethu S.R., Kwon R.S., Mamula P., Pedrosa M.C., ASGE Technology Committee (2010). Interventional EUS. Gastrointest. Endosc..

[14] Devière J. (2023). Endoscopic Ultrasound-Guided Pancreatic Duct Interventions. Gastrointest. Endosc. Clin. N. Am..

[15] Sobani Z.A., Ling C., Rustagi T. (2021). Endoscopic Ultrasound-Guided Gallbladder Drainage. Dig. Dis. Sci..

[16] Hashimoto R., Chang K.J. (2021). Endoscopic ultrasound guided hepatic interventions. Dig. Endosc..

[17] Ebbehøj N., Borly L., Bülow J., Grønvall S., Madsen R.P., Matzen P., Øwre A. (1990). Pancreatic tissue fluid pressure in chronic pancreatitis relation to pain, morphology, and function. Scand. J. Gastroenterol..

[18] Tessier G., Bories E., Arvanitakis M., Hittelet A., Pesenti C., Le Moine O., Giovannini M., Devière J. (2007). EUS-guided pancreatogastrostomy and pancreatobulbostomy for the treatment of pain in patients with pancreatic ductal dilatation inaccessible for transpapillary endoscopic therapy. Gastrointest. Endosc..

[19] Jimenez R.E., Castillo C.F., Rattner D.W., Warshaw A.L. (2003). Pylorus-preserving pancreaticoduodenectomy in the treatment of chronic pancreatitis. World J. Surg..

[20] Beger H.G., Schlosser W., Friess H.M., Büchler M.W. (1999). Duodenum-preserving head resection in chronic pancreatitis changes the natural course of the disease: A single-center 26-year experience. Ann. Surg..

[21] Cahen D.L., Gouma D.J., Nio Y., Rauws E.A., Boermeester M.A., Busch O.R., Stoker J., Laméris J.S., Dijkgraaf M.G., Huibregtse K. (2007). Endoscopic versus surgical drainage of the pancreatic duct in chronic pancreatitis. N. Engl. J. Med..

[22] Cremer M., Devière J., Delhaye M., Baize M., Vandermeeren A. (1991). Stenting in severe chronic pancreatitis: Results of medium-term follow-up in seventy-six patients. Endoscopy.

[23] Gabbrielli A., Pandolfi M., Mutignani M., Spada C., Perri V., Petruzziello L., Costamagna G. (2005). Efficacy of main pancreatic-duct endoscopic drainage in patients with chronic pancreatitis, continuous pain, and dilated duct. Gastrointest. Endosc..

[24] Rösch T., Daniel S., Scholz M., Huibregtse K., Smits M., Schneider T., Ell C., Haber G., Riemann J.-F., Jakobs R. (2002). Endoscopic treatment of chronic pancreatitis: A multicenter study of 1000 patients with long-term follow-up. Endoscopy.

[25] Delhaye M., Arvanitakis M., Verset G., Cremer M., Devière J. (2004). Long-term clinical outcome after endoscopic pancreatic ductal drainage for patients with painful chronic pancreatitis. Clin. Gastroenterol. Hepatol..

[26] Mallery S., Matlock J., Freeman M.L. (2004). EUS-guided rendezvous drainage of obstructed biliary and pancreatic ducts: Report of 6 cases. Gastrointest. Endosc..

[27] Will U., Fueldner F., Thieme A.-K., Goldmann B., Gerlach R., Wanzar I., Meyer F. (2007). Transgastric pancreatography and EUS-guided drainage of the pancreatic duct. J. Hepato-Biliary-Pancreatic Surg..

[28] Kahaleh M., Hernandez A.J., Tokar J., Adams R.B., Shami V.M., Yeaton P. (2007). EUS–guided pancreaticogastrostomy: Analysis of its efficacy to drain inaccessible pancreatic ducts. Gastrointest. Endosc..

[29] Tyberg A., Sharaiha R.Z., Kedia P., Kumta N., Gaidhane M., Artifon E., Giovannini M., Kahaleh M. (2017). EUS-guided pancreatic drainage for pancreatic strictures after failed ERCP: A multicenter international collaborative study. Gastrointest. Endosc..

[30] Imoto A., Ogura T., Higuchi K. (2020). Endoscopic Ultrasound-Guided Pancreatic Duct Drainage: Techniques and Literature Review of Transmural Stenting. Clin. Endosc..

[31] Sakai T., Koshita S., Kanno Y., Ogawa T., Kusunose H., Yonamine K., Miyamoto K., Kozakai F., Okano H., Ohira T. (2022). Early and long-term clinical outcomes of endoscopic interventions for benign pancreatic duct stricture/obstruction-the possibility of additional clinical effects of endoscopic ultrasonography-guided pancreatic drainage-. Pancreatology.

[32] Dalal A., Patil G., Maydeo A. (2020). Six-year retrospective analysis of endoscopic ultrasonography-guided pancreatic ductal interventions at a tertiary referral center. Dig. Endosc..

[33] Bhurwal A., Tawadros A., Mutneja H., Gjeorgjievski M., Shah I., Bansal V., Patel A., Sarkar A., Bartel M., Brahmbhatt B. (2021). EUS guided pancreatic duct decompression in surgically altered anatomy or failed ERCP—A systematic review, meta-analysis and meta-regression. Pancreatology.

[34] Matsunami Y., Itoi T., Sofuni A., Tsuchiya T., Kamada K., Tanaka R., Tonozuka R., Honjo M., Mukai S., Fujita M. (2018). Evaluation of a new stent for EUS-guided pancreatic duct drainage: Long-term follow-up outcome. Endosc. Int. Open.

[35] Shimizu H., Suzuki R., Sato Y., Takagi T., Abe N., Irie H., Sugimoto M., Yanagita T., Kobashi R., Hashimoto M. (2022). Transjejunal endoscopic ultrasound-guided pancreatic drainage for pancreatic jejunostomy stricture using a forward-viewing echoendoscope in a patient with altered anatomy. DEN Open.

[36] Teh J.L., Teoh A.Y.B. (2023). Techniques and Outcomes of Endoscopic Ultrasound Guided—Pancreatic Duct Drainage (EUS-PDD). J. Clin. Med..

[37] Ergun M., Aouattah T., Gillain C., Gigot J.-F., Hubert C., Deprez P.H. (2011). Endoscopic ultrasound-guided transluminal drainage of pancreatic duct obstruction: Long-term outcome. Endoscopy.

[38] Chen Y.-I., Levy M.J., Moreels T.G., Hajijeva G., Will U., Artifon E.L., Hara K., Kitano M., Topazian M., Abu Dayyeh B. (2017). An international multicenter study comparing EUS-guided pancreatic duct drainage with enteroscopy-assisted endoscopic retrograde pancreatography after Whipple surgery. Gastrointest. Endosc..

[39] Van Der Merwe S.W., Van Wanrooij R.L., Bronswijk M., Everett S., Lakhtakia S., Rimbas M., Hucl T., Kunda R., Badaoui A., Law R. (2022). Therapeutic endoscopic ultrasound: European Society of Gastrointestinal Endoscopy (ESGE) Guideline. Endoscopy.

[40] Jearth V., Sundaram S., Kale A., Sachan A., Rana S.S. (2024). Current paradigm of endoscopic ultrasound in biliary and pancreatic duct drainage: An update. Ann. Gastroenterol..

[41] Brauer B.C., Chen Y.K., Fukami N., Shah R.J. (2009). Single-operator EUS-guided cholangiopancreatography for difficult pancreaticobiliary access (with video). Gastrointest. Endosc..

[42] Barkay O., Sherman S., McHenry L., Yoo B.M., Fogel E.L., Watkins J.L., DeWitt J., Al-Haddad M.A., Lehman G.A. (2010). Therapeutic EUS-assisted endoscopic retrograde pancreatography after failed pancreatic duct cannulation at ERCP. Gastrointest. Endosc..

[43] Shah J.N., Marson F., Weilert F., Bhat Y.M., Nguyen-Tang T., Shaw R.E., Binmoeller K.F. (2012). Single-operator, single-session EUS-guided anterograde cholangiopancreatography in failed ERCP or inaccessible papilla. Gastrointest. Endosc..

[44] Vila J.J., Pérez-Miranda M., Vazquez-Sequeiros E., Abadia M.A.-S., Pérez-Millán A., González-Huix F., Gornals J., Iglesias-Garcia J., De la Serna C., Aparicio J.R. (2012). Initial experience with EUS-guided cholangiopancreatography for biliary and pancreatic duct drainage: A Spanish national survey. Gastrointest. Endosc..

[45] Kurihara T., Itoi T., Sofuni A., Itokawa F., Moriyasu F. (2013). Endoscopic ultrasonography-guided pancreatic duct drainage after failed endoscopic retrograde cholangiopancreatography in patients with malignant and benign pancreatic duct obstructions. Dig. Endosc..

[46] Fujii L.L., Topazian M.D., Abu Dayyeh B.K., Baron T.H., Chari S.T., Farnell M.B., Gleeson F.C., Gostout C.J., Kendrick M.L., Pearson R.K. (2013). EUS-guided pancreatic duct intervention: Outcomes of a single tertiary-care referral center experience. Gastrointest. Endosc..

[47] Will U., Füldner F., Reichel A., Meyer F. (2014). [EUS-guided drainage of the pancreatic duct (EUPD)—Promising therapeutic alternative to surgical intervention in case of symptomatic retention of the pancreatic duct and unsuccessful ERP]. Zentralbl. Chir..

[48] Oh D., Park D.H., Cho M.K., Nam K., Song T.J., Lee S.S., Seo D.-W., Lee S.K., Kim M.-H. (2016). Feasibility and safety of a fully covered self-expandable metal stent with antimigration properties for EUS-guided pancreatic duct drainage: Early and midterm outcomes (with video). Gastrointest. Endosc..

[49] Uchida D., Kato H., Saragai Y., Takada S., Mizukawa S., Muro S., Akimoto Y., Tomoda T., Matsumoto K., Horiguchi S. (2018). Indications for Endoscopic Ultrasound-Guided Pancreatic Drainage: For Benign or Malignant Cases?. Can. J. Gastroenterol. Hepatol..

[50] Oh D., Park D.H., Song T.J., Lee S.S., Seo D., Lee S.K., Kim M. (2020). Long-term outcome of endoscopic ultrasound-guided pancreatic duct drainage using a fully covered self-expandable metal stent for pancreaticojejunal anastomosis stricture. J. Gastroenterol. Hepatol..

[51] Chandan S., Mohan B.P., Khan S.R., Kassab L.L., Ponnada S., Ofosu A., Bhat I., Singh S., Adler D.G. (2020). Efficacy and safety of endoscopic ultrasound-guided pancreatic duct drainage (EUS-PDD): A systematic review and meta-analysis of 714 patients. Endosc. Int. Open.

[52] Boicean A., Birlutiu V., Ichim C., Todor S.B., Hasegan A., Bacila C., Solomon A., Cristian A., Dura H. (2023). Predictors of Post-ERCP Pancreatitis (P.E.P.) in Choledochal Lithiasis Extraction. J. Pers. Med..

[53] Boicean A., Prisca D., Bratu D.G., Bacila C.I., Tanasescu C., Chicea R., Fleaca S.R., Birsan S.A., Ichim C., Mohor C.I. (2024). Uncommon Presentation of Gastric Duplication Cyst with Left-Sided Portal Hypertension: A Case Report and Literature Review. Diagnostics.

[54] Cui Y., Kozarek R.A. (2023). Evolution of Pancreatic Endotherapy. Gastrointest. Endosc. Clin. N. Am..

[55] Vitali F., Zundler S., Jesper D., Strobel D., Wildner D., de Pretis N., Frulloni L., Crinó S.F., Neurath M.F. (2023). Endoscopic Ultrasound in Pancreatology: Focus on Inflammatory Diseases and Interventions. Visc. Med..

[56] Sorrentino L., Chiara O., Mutignani M., Sammartano F., Brioschi P., Cimbanassi S. (2017). Combined totally mini-invasuve approach in nectrotizing pancreatitis: A case report and systematic literature review. World J. Emerg. Surg..

[57] Sion M.K., A Davis K. (2019). Step-up approach for the management of pancreatic necrosis: A review of the literature. Trauma. Surg. Acute Care Open.

[58] Gupta R., Kulkarni A., Babu R., Shenvi S., Gupta R., Sharma G., Kang M., Gorsi U., Rana S.S. (2020). Complications of percutaneous Drainage in Step-up-Approach for Management of Pancreatic Necrosis: Experience of 10 Years. J. Gastroint. Surg..

[59] Yasuda I., Takahashi K. (2020). Endoscopic management of walled-off pancreatic necrosis. Dig. Endosc..

[60] van Brunschot S., van Grinsven J., van Santvoort H.C., Bakker O.J., Besselink M.G., Boermeester M.A., Bollen T.L., Bosscha K., Bouwense S.A., Bruno M.J. (2018). Endoscopic or surgical step-up approach for infected necrotising pancreatitis: A multicentre randomized trial. Lancet.

[61] Wundsam H.V., Spaun G.O., Bräuer F., Schwaiger C., Fischer I., Függer R. (2019). Evolution of Transmural Necrosectomy for Acute Pancreatitis to Stent in Stent Therapy: Step-up Approach Leads to Low Mortality and Morbidity Rates in 302 Consecutive Cases of Acute Pancreatitis. J. Laparoendosc. Adv. Surg. Tech. A.

[62] Bang J.Y., Arnoletti J.P., Holt B.A., Sutton B., Hasan M.K., Navaneethan U., Feranec N., Wilcox C.M., Tharian B., Hawes R.H. (2019). An endoscopic transluminal approach, compared with minimally invasive surgery, reduces complications and costs for patients with necrotizing pancreatitis. Gastroenterology.

[63] Giovannini M. (2022). EUS-guided transenteric pancreatic duct drainage. Best. Pract. Res. Clin. Gastroenterol..

